# Supplementary material for: Lightweight and drift-free magnetically actuated millirobots via asymmetric laser-induced graphene
Source: Nat Commun. 2024 May 21;15:4334. doi: 10.1038/s41467-024-48751-x (PMC11109242; doi:10.1038/s41467-024-48751-x)
Supplement: Supplementary file 1 — Supplementary Information [file 41467_2024_48751_MOESM1_ESM.pdf]

Supplementary information for

Lightweight and drift-free magnetically actuated millirobots via asymmetric laser-induced graphene

Yun Chen[1], Yuanhui Guo[1], Bin Xie[1], Fujun Jin[2], Li Ma[1], Hao Zhang[1], Yihao Li[3], Xin Chen[1]*, Maoxiang Hou[1], Jian Gao[1], Huilong Liu[1], Yu-Jing Lu[2]*, Ching-Ping Wong[4], Ni Zhao[3]*

[1] State Key Laboratory of Precision Electronic Manufacturing Technology and Equipment, School of Electromechanical Engineering, Guangdong University of Technology, Guangzhou, 510006, PR China

E-mail: chenx@gdut.edu.cn

[2] Institute of Natural Medicine and Green Chemistry, School of Biomedical and Pharmaceutical Sciences, Guangdong University of Technology, Guangzhou, 510006, PR China.

E-mail: luyj@gdut.edu.cn

[3] Department of Electronic Engineering, The Chinese University of Hong Kong, Shatin, Hong Kong, China

E-mail: nzhao@ee.cuhk.edu.hk

[4] School of Materials Science and Engineering, Georgia Institute of Technology, Atlanta, Georgia, 30332, United States of America.

Supplementary information list:

Note S1. Material-dependent millirobot motion

Note S2. Drug release of GM-Millirobot

Note S3. Cost-effectiveness of each GH millirobot

Fig. S1. Principle of shaping the laser beam

Fig. S2. Raman and wetting property characterizations of LIG sheet

Fig. S3. Schematic diagram of a LIG sheet processed by the circular laser spot

Fig. S4. Ablation shapes of defocused laser spots on PI film

Fig. S5. Batch processing for the porous helical LIG sheets

Fig. S6. Effects of laser processing parameters on the geometrial configurations of the helical LIG sheet

Fig. S7. Porous helical LIG sheets with sputtered metal Ni

Fig. S8. Elemental composition on the surface of GH millirobot

Fig. S9. Magnetic driving system used in the experiment

Fig. S10. Processing high-density GH millirobots

Fig. S11. Rotation of GH millirobots with right-handed (blue) and left-handed (red) chirality under the same magnetic field

Fig. S12. Time-lapse photographs of the motion of a DOX-HCl@GH millirobot, controlled by a rotating magnetic field of 12 mT, in an isolated porcine bladder

Fig. S13. Safety verification of GH millirobot for living mice

Fig. S14. Safety verification of GH millirobot for living mice: the routine hematological analysis and the biopsies H&E staining of organs

Fig. S15. Safety verification of GH millirobot for living mice: biochemistry analysis

Fig. S16. Mice tumor therapy

Fig. S17. Routine hematological analysis of mice tumor therapy

Table S1. Comparison of swimming speeds and lateral drift rates of millirobots of conventional materials

Note S1: Material-dependent millirobot motion

The motion of a magnetically actuated millirobot can be described using a speed vector $\vec{U}$ $(U_x, U_y\ U_z)$, where U_x is the rate of lateral drift due to near-wall effects and wall-induced friction, U_y is the rate of propulsion induced by the rotating magnetic field, and U_z is the rate of sinking, if gravity dominates over buoyancy.

For a C-Millirobot, as there is a certain distance between the millirobot and the bottom wall (h>>0) at the initial, the near-wall effect is avoided. Therefore, the motion of the millirobot can be categorized as motion far away from bottom wall which can be subdivided into the sinking movement (U_z) due to the imbalance between gravity and buoyancy, and the propulsion (U_y) induced by the magnetic torque resulted from the rotating magnetic field. While millirobots is near the bottom wall (h≈0), its motion is categorized as motion near the bottom wall which can be divided into two parts as well, including the lateral drift (U_x) due to near-wall effects and friction between the millirobot and the bottom wall, and the propulsion (U_y) driven by the magnetic driving torque of the rotating magnetic field.

Each motion of the C-Millirobot can be given as follows:

$$\overrightarrow{U_x} = \frac{1}{C_{x1}} e^{-\frac{H}{m}t} + \int_{t_0}^{t} e^{\frac{H}{m}(\tau - t)} \frac{\overrightarrow{f_r} - \overrightarrow{f_{n-w}}}{m} d\tau \qquad h = 0 \tag{1}$$

$$\overrightarrow{U_y} = R\omega Ch\left[1 - \frac{2}{1+\gamma^2}\frac{\omega_{s-o}^2}{\omega^2}\left(1 + \sqrt{1 - \frac{\omega^2}{\omega_{s-o}^2}}\right)\right] \qquad h \geq 0 \tag{2}$$

$$\overrightarrow{U_z} = \frac{1}{C_{z1}} e^{-\frac{\xi}{m}t} + \int_{t_0}^{t} e^{\frac{\xi}{m}(\tau - t)} \frac{\overrightarrow{f_b} + \vec{G}}{m} d\tau \qquad h \gg 0 \tag{3}$$

where C_{x1} and C_{z1} are constants; H is the friction coefficient between the millirobot and the bottom wall; Ch and γ are the chiral coefficient and the "steerability" parameter, respectively, which are both related to the geometry of the millirobot; ξ is the viscosity coefficient related to the properties of the liquid and the geometry of the millirobot.

The aforementioned paradigm can be altered by reducing the density of the millirobot so that the buoyant force is greater than the gravitational force. In this case, the millirobot maintains floating and the wall effects are negligible, resulting in close-to-zero rates in both the x- and z-directions, i.e., the rate vector becomes $(0, U_y, 0)$ and a straight trajectory can be obtained. Considering a liquid density of 0.998 g/cm^3 (assuming the liquid is deionised water), the density of the millirobot should ideally be below 1.000 g/cm^3 to reach the float state. On the other hand, to enable magnetic field control, a high enough loading of magnetic nanoparticles is required. Here, we use nickel nanoparticles, which have a bulk density of 8.902g/cm^3, and take the volume fraction (ϕ) of nanoparicles[58] as $\phi = 0.06$, the average density of the millirobot, $\rho_{microrobot}$, can be calculated as $\rho_{microrobot} = \rho_{scaffold}(1 - \phi) + \rho_{Ni} * \phi$. Accordingly, the density of the helical scaffold $(\rho_{scaffold})$ must have a density below 0.5 g/cm^3

to render the millirobot floating in the liquid. Such a low density can be obtained from highly porous structures composed of light elements. As such, porous graphene becomes our choice of material for creating the helical scaffold.

In this case, the millirobot maintains floating and the wall effects are negligible, resulting in close-to-zero rates in both the x- and z-directions, i.e., the rate vector becomes $(0, U_y, 0)$ and a straight trajectory can be obtained.

Thus for a GH millirobot, because of the balance between the gravity and the buoyancy, each axis of the GH millirobot is only affected by the magnetic driving torque and the torque induced by the viscous force. Thus, each motion of the GH millirobot can be given as:

$$\vec{U} = \overrightarrow{U_y} = R\omega Ch\left[1 - \frac{2}{1+\gamma^2}\frac{\omega_{s-o}^2}{\omega^2}\left(1 + \sqrt{1 - \frac{\omega^2}{\omega_{s-o}^2}}\right)\right] \qquad h \geq 0 \tag{4}$$

The GH millirobot steps out when the magnetic field's magnetic torque is not able to maintain a synchronous relationship between the magnetic moment and the applied rotating magnetic field. Therefore, the geometry of the helix, the viscosity of the liquid, the magnetic materials characteristics and the strength of the magnetic field are the main factors that influence the step-out frequency, which can be formulated as[57]:

$$\omega_{s-o} = \omega^* \frac{1+\gamma^2}{2\gamma} = \frac{\Delta\chi H^2 V}{\kappa_\perp} sin2\Phi \tag{5}$$

where $\Delta\chi$ and H are effected by saturation magnetization and magnetic materials, respectively, and Φ is the angle between helical axis and axis of rotating magnetic field. The forward swimming velocity after step out can be predicted by the formula as[58]:

$$\overrightarrow{U_y} = A\omega Ch\left[1 - \frac{2}{1+\gamma^2}\frac{\omega_{s-o}^2}{\omega^2}\left(1 + \sqrt{1 - \frac{\omega^2}{\omega_{s-o}^2}}\right)\right](1 - \frac{\sqrt{\omega^2 - \omega_{s-o}^2}}{\omega}) \tag{6}$$

The normalizing radiu $A = 2\pi r$, where r is the rediu of GH millirobot. The “steerability” parameter γ used in prediction curve are 15.76, 11.10, 9.49, 8.26, 6.60. And the chirality coefficients Ch used in prediction curve are 0.185, 0.169, 0.16, 0.153, 0.147 depending on the geometric parameters of GH millirobot, respectively.

Note S2. Drug release of GM-Millirobot

To verify whether the drug release of each GH millirobot was consistent among different batches, we randomly selected ten GH millirobots from Batch 1 and five GH millirobots from Batch 2, respectively, for comparison (Fig.5d). Under the same NIR irradiation intensity, the ten GH millirobots from Batch 1 released 0.32 μg of drug in total and the five GH millirobots from batch 2 released 0.15 μg of drug in total, both to 2 mL of deionized water in 5 min, corresponding to an average release amount of 0.032 μg per GH millirobot for Batch 1 and 0.030 μg per GH millirobot for Batch 2. The results confirm that the drug release performance of the GH millirobots is consistent among different batches.

Besides, to characterize the fall-off of the loaded drugs, we monitored the drug concentration in the liquid environment where the DOX-HCl-loaded GH millirobots were suspended for 90 minutes. As shown by the green curve in Fig. 5d, only 0.188 μg DOX-HCl was dislodged from a total of ten GM-Millirobots after the 90-min movement. Note that the GH millirobots can move very fast with a maximum speed of 3.1 mm/s, thus they can reach the target position in a very short time (usually less than thirty seconds). Therefore, there will be extremely little drug dislodged during the delivery process.

Note S3. Cost-effectiveness of each GH millirobot

The dominant capital cost of setting up our GH millirobot processing system is the cost of the UV picosecond laser (~US＄430,000 including maintenance) and the magnetron sputtering machine (~US＄28,000 including maintenance); which all have a limited lifetime of ~ 10,000 hours. Considering that the GH millirobots can be fabricated at a speed of 77 scaffolds per second, and then sputtering-coated with Ni at a batch load of 6,000 scaffolds per hour, and including the cost of PI tapes, the average processing cost of each GH millirobot could be as low as ~＄0.0007.

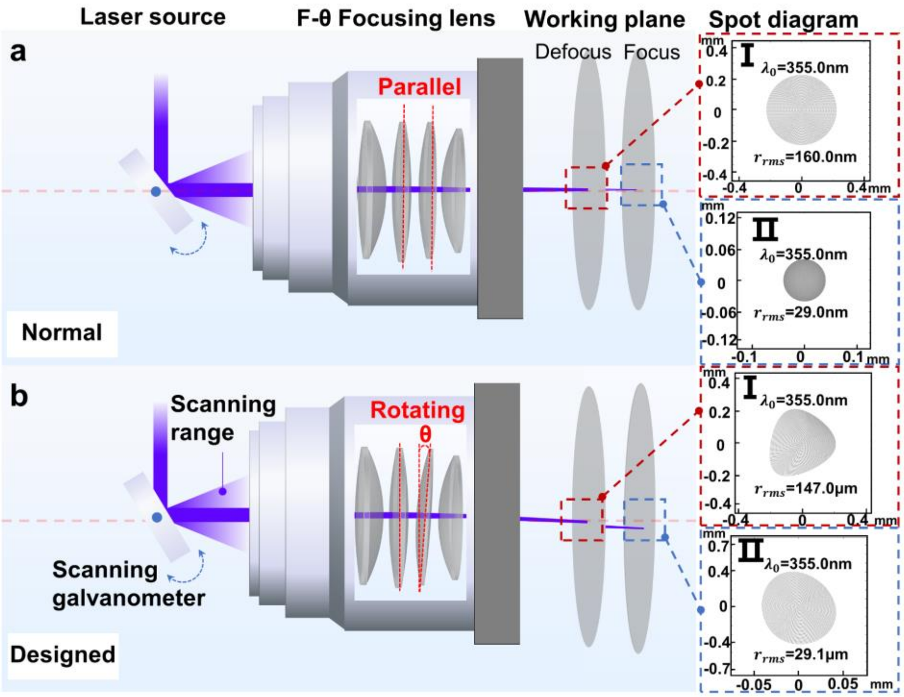

Fig. S1. Principle of shaping the laser beam. a, In a conventional setting, the deflected laser beam is focused to a circular spot at the working plane, so the spot on the defocus plane I is simply the magnification of that on the focus plane II. **b,** By deliberately tilting one of the F-θ lenses, the laser beam is shifted away from the original focusing plane, and introducing asymmetric distortions which are magnificated by the defocuse distance to the laser spot on the working plane.

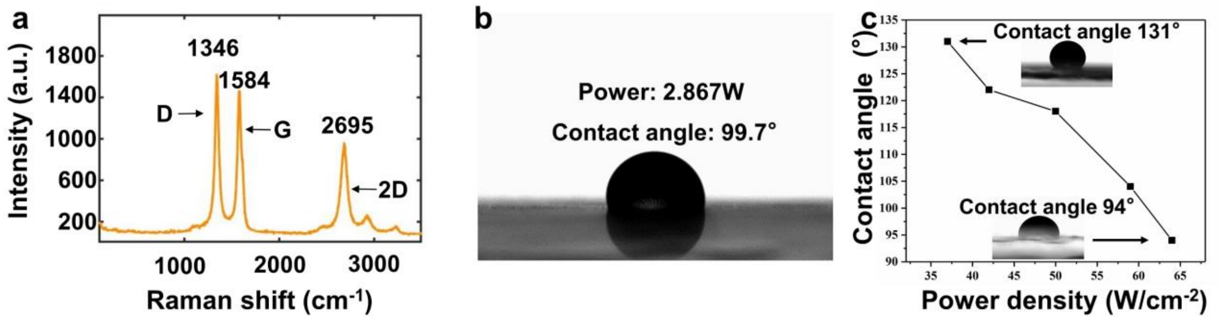

Fig. S2. Raman and wetting property characterizations of LIG sheet. a, The Raman spectrum demonstrates that the characteristic peaks of LIG sheets include the D (~1350 cm^{-1}), G (~1584 cm^{-1}) and 2D (~2695 cm^{-1}) peaks, which indicates that the sheets are indeed composed of graphene. **b,** Hydrophobicity test of LIG sheet. **c**, Effects of power density on the surface wettability.

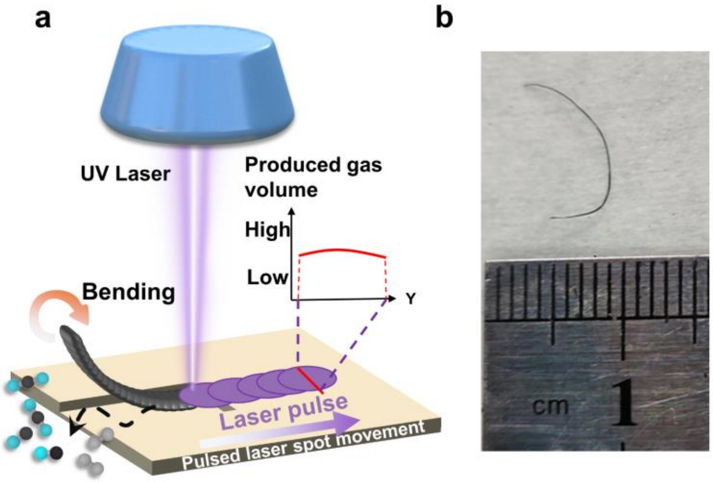

Fig. S3. Schematic diagram of a LIG sheet processed by a circular laser spot. a, When the laser spot is circular, the gas formation along the scanning line is uniform, therefore, only a bended LIG sheet is formed. **b,** Optical image of a LIG sheet processed by the circular laser spot.

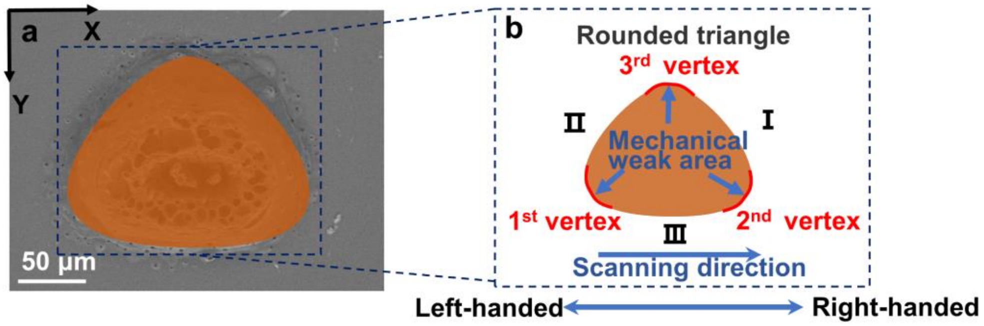

Fig. S4. Ablation shapes of defocused laser spots on PI film. a, After defocused processing a point using the laser, the laser-generated ablation marks (LIG micro-dot) on the PI film approximate a rounded triangle. **b,** The rounded triangle consists of three edges and three rounded vertices (mechanically weak regions). In the mechanically weak region, the LIG microdots begin to separate from the PI film. As the pulsed laser moves, LIG micro-dots are generated continually and then connected into LIG sheets. At the same time, the LIG sheet start to separate from the PI film at the region of mechanical weakness (the region away from the laser scanning direction) and twist in the same direction. After finishing and fixing, the twisted LIG sheets eventually form a porous helical LIG sheet with uniform spacing and diameter. Different laser scanning directions enable the fabrication of spiral LIG sheets with a controlled rotational direction.

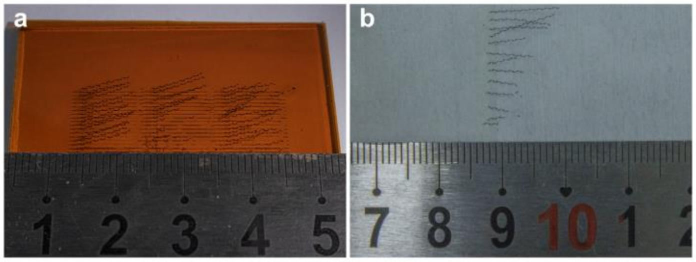

Fig. S5. Batch processing for the porous helical LIG sheets. a, The helical LIG sheets were highly reproducible and **b** had controllable lengths.

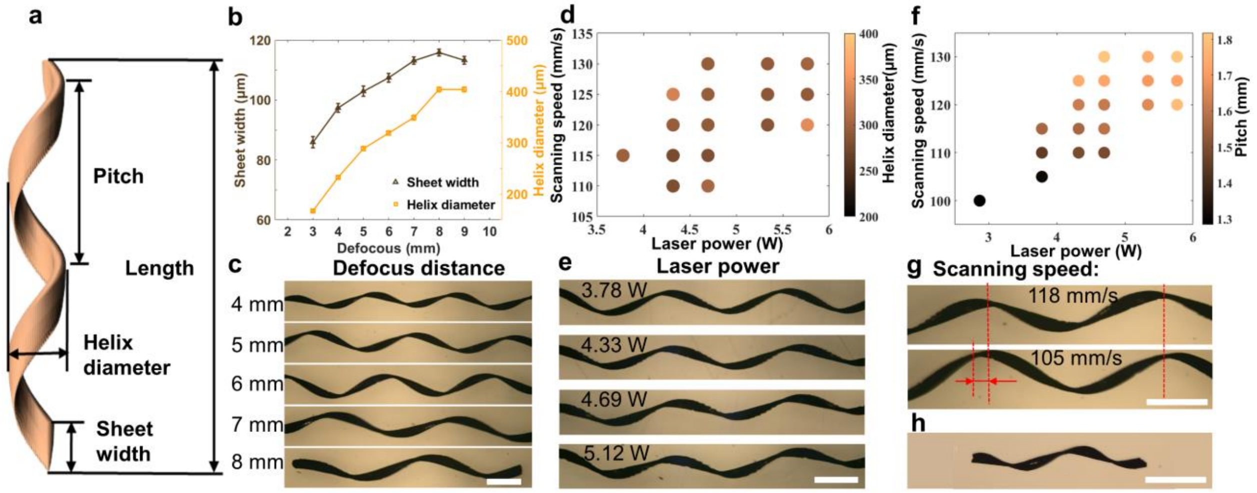

Fig. S6. Effects of laser processing parameters on the geometrical configurations of the helical LIG millisheet. a, Geometric configuration definition of the helical LIG millisheet. **b**, Effects of defocus distance on the helix diameter and sheet width and **c** the corresponding optical microscope images. When the defocus distance increased from 3 mm to 9 mm, the sheet width increased from 86 ± 4 μm to the largest value of 117 ± 3 μm at 8 mm and then slightly decreased to 110 ± 6 μm, the helix diameter correspondingly increased from 167 ± 4 μm to 498 ± 10 μm. **d,** Effects of laser power and scanning speed on the helix diameter and **e** the corresponding optical microscope image. The laser power and scanning speed had minor effects on the helix diameter of the helical LIG sheets. The helix diameter was within the range of 300 ± 20 μm with a deviation of less than 6.7% under different laser powers. The helix diameter varied no more than ± 10 μm with a deviation within a range of less than 3.4% under different scanning speeds. **f,** Effect of laser power and scanning speed on the helix pitch and **g** the corresponding optical microscope image. Laser power had little effect on the helix pitch: when the laser power was varied from its minimum to maximum values, the helix pitch varied no more than ± 20 μm (i.e. with less than 1.3% variation). When the scanning speed increased from 85 mm/s to 130 mm/s, the helix pitch increased from 986 ± 6 μm to 1792 ± 20 μm. **h,** Smallest millirobot processed at a defocus distance of 3 mm, a scanning speed of 85 mm/s, and a laser power of 2 W. Scale bars: 500 μm.

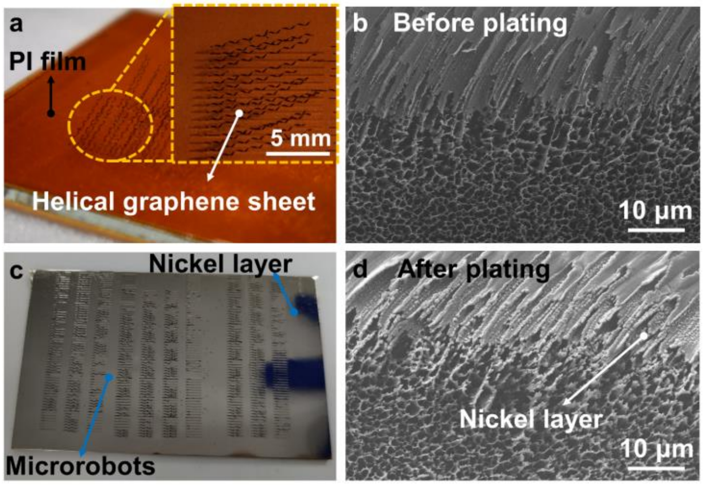

Fig. S7. Porous helical LIG sheets with sputtered metal Ni. a, The pristine porous helical LIG sheet induced from amber PI film and **b** its surface structure. **c**, After sputtering a metallic nickel layer on helical LIG sheets, their surface color converted to a silvery white with a metallic sheen and **d** SEM image shows dense nickel nanoparticles were loaded on the surface.

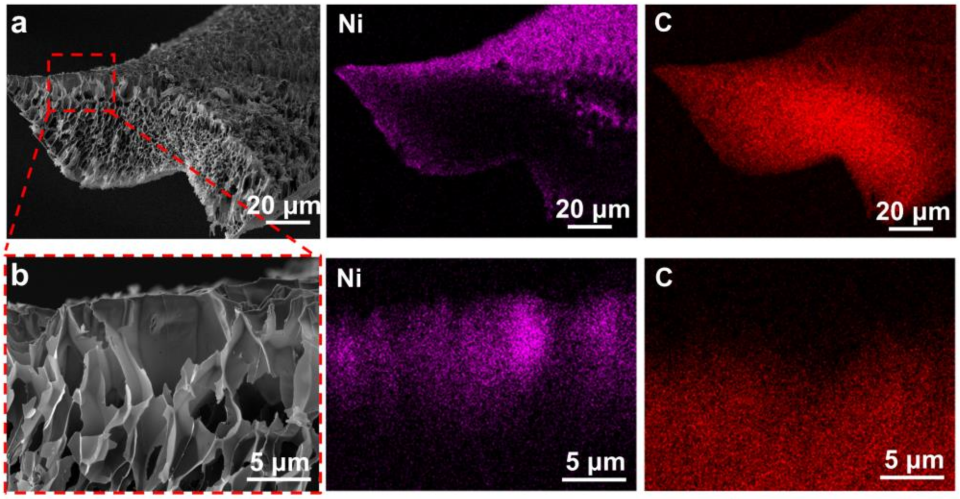

Fig. S8. **Elemental composition on the surface of GH millirobot. a,** The SEM-EDS mappings of the GH-Miccrorobots cross-section shows carbon and nickel are the main elements distributed on the surface of the LIG sheet. Scale bar: 20 μm. **b,** The SEM-EDS mappings at a larger magnification shows the same distrbutions. Scale bar: 5 μm.

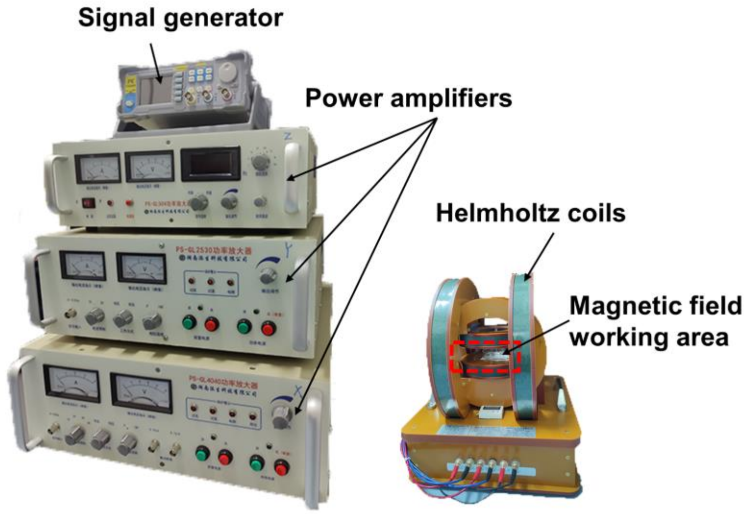

Fig. S9. **Magnetic driving system.** Magnetic driving system used in the experiment, includes the signal generator, power amplifiers and Helmholtz coils. The combined effect of these components creates a uniformly strong rotating magnetic field within its working area.

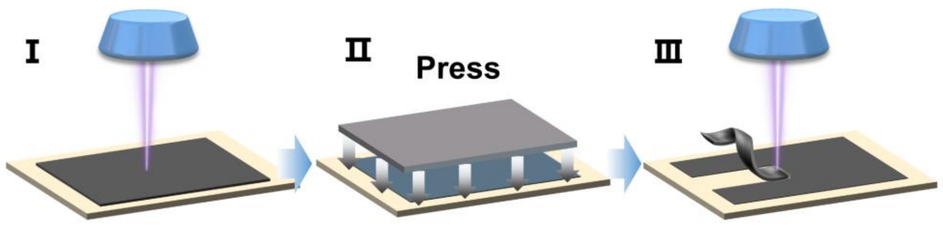

Fig. S10. Processing high-density GH millirobots. Firstly, a planar LIG sheet was processed with a laser power density of 35 J/cm^2; subsequently, a pressure of 10 tons was applied to the planar LIG sheet for 10 min. Then the resulted high density planar LIG sheet was processed using the same laser parameters used in the processing the low-density one, resulting in a high density helical LIG sheet. After that, it was coated with nickel resulting in a density of approximately 1.42 g/cm^3.

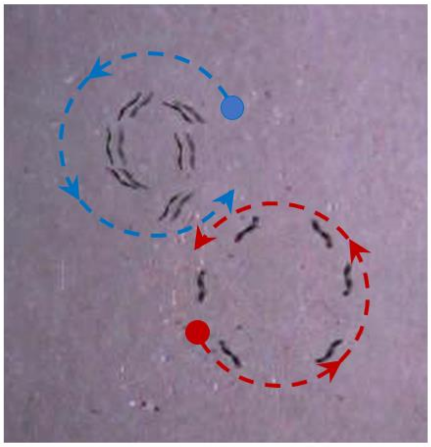

Fig. S11. Rotation of GH millirobots with right-handed (blue) and left-handed (red) chirality under the same magnetic field.

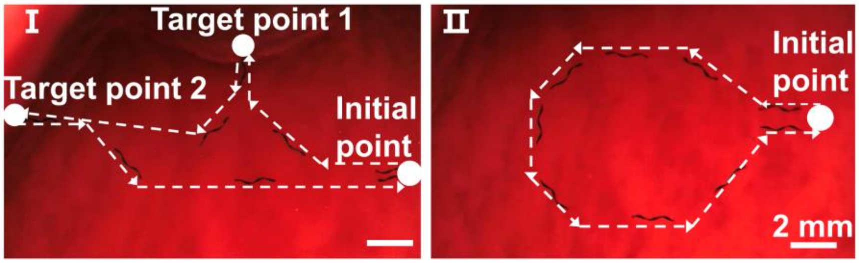

Fig. S12. Time-lapse photographs of the motion of a DOX-HCl@GH millirobot, controlled by a rotating magnetic field of 12 mT, in an isolated porcine bladder. **I** The GH millirobot was navigated from the initial point to target points 1 and 2 sequentially, adhering to a predetermined trajectory designed to maintain distance from the inner wall of the bladder. **II** The GH millirobot performed multi-angle manipulation around a circular path within the isolated pig bladder, showcasing the advantageous characteristics of its low density, which enabled full suspension and flexible adjustment of movement angles.

Notes:Throughout these maneuvers, the microrobot effectively maintained a safe distance from the mucosal surface of the bladder wall, thus circumventing potential interference from surface structures—a common challenge encountered in the operation of wall-contact microrobots. Of significant importance, this avoidance of wall contact ensures the protection of non-cancerous cells from exposure to drugs during transport. These demonstrations underscore the advantages of GH millirobots in executing prolonged locomotion and facilitating drug transport within a physiological environment.

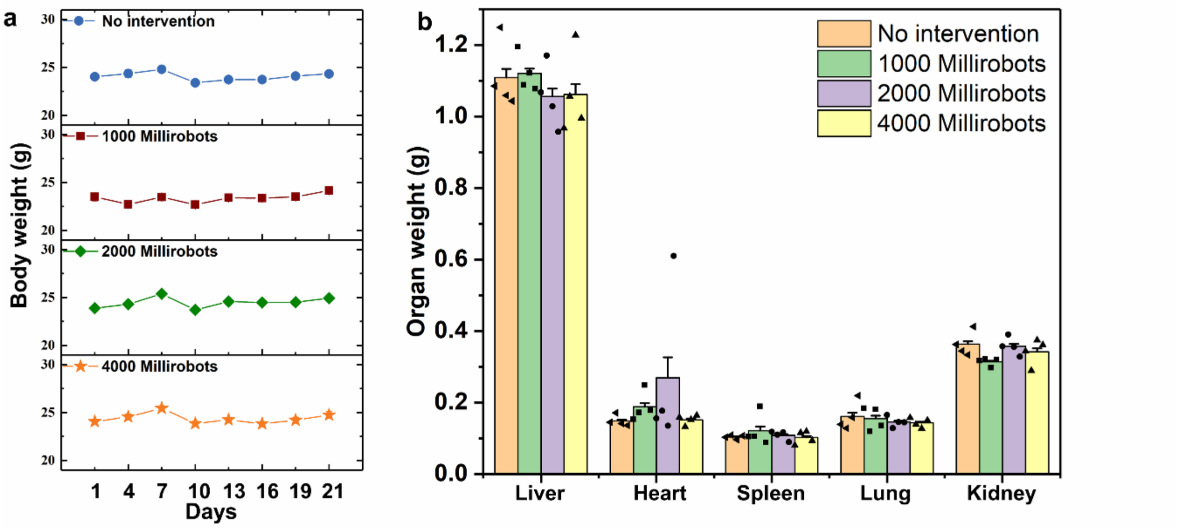

Fig. S13. Safety examination of GH millirobots on living mice. a, Body weight of mice during 7 consecutive days gavage of millirobots (control group, mice in other groups were in gavages of 1000, 2000 and 4000 GH millirobot, respectively) and follow-up 14 days monitoring, (n=4/group). **b,** Comparison of organ weights of mice at the end of the safety experiment, (n=4/group). Data are presented as the means ± s.e.m.

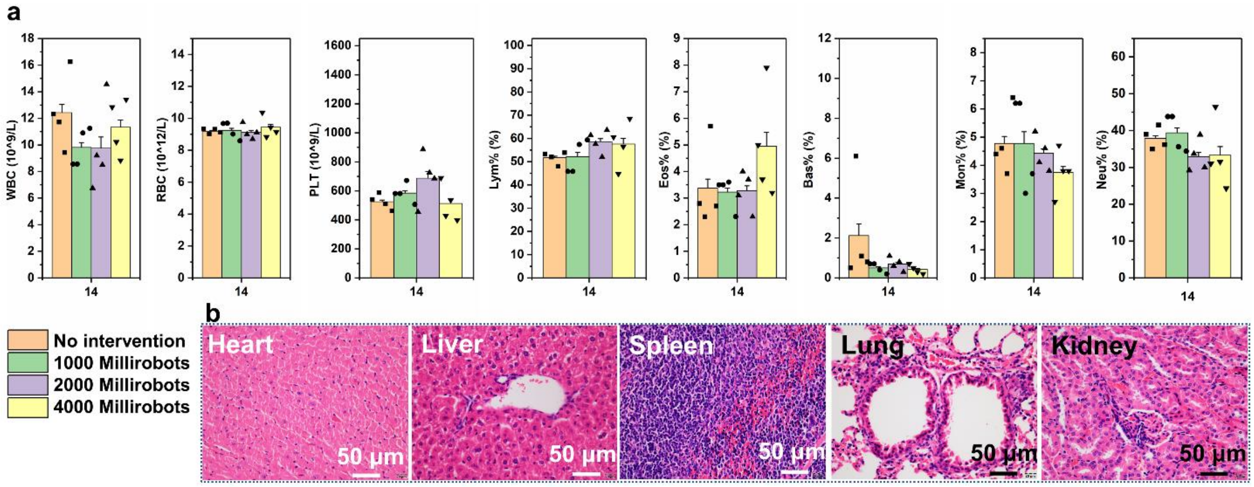

Fig. S14. Safety examination of GH millirobots in living mice: routine hematological analysis and biopsies H&E staining of organs. a, Blood routine examination results of mice at 14th day. There were different numbers of pristine GH millirobots by gavage in mice and the safety test performed continuously for 7 days at a volume of 0.2 mL per mice in each day, and all the mice were continuously observed for 14 days, (n=4/group). Data are presented as the means ± s.e.m. **b,** Tissues' biopsies (including heart, liver, spleen, lung, kidney) for H&E staining, which were collected from group iv (4000 GH millirobots) in safety experiments.

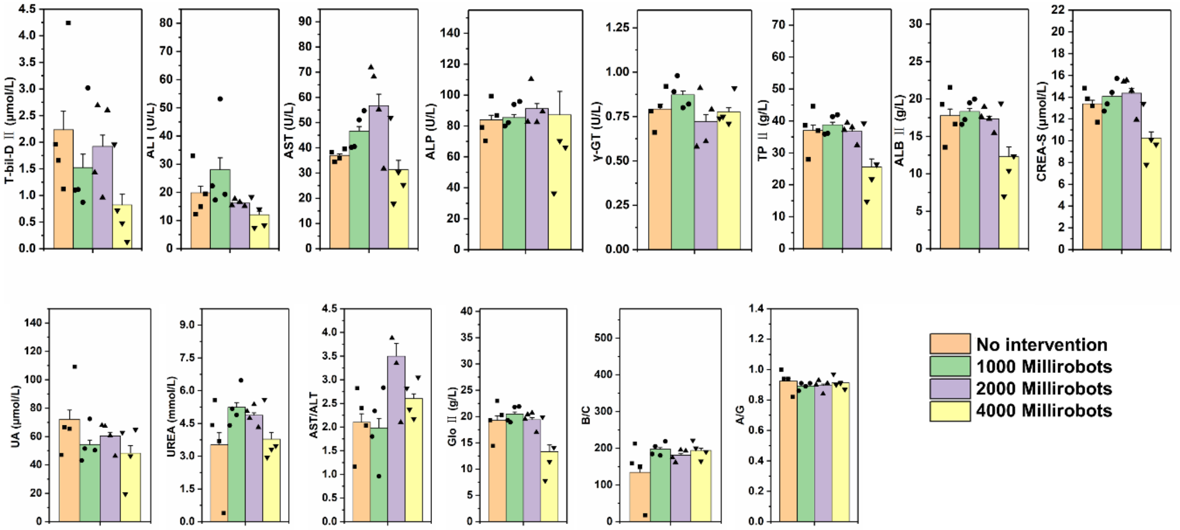

Fig. S15. Safety examination of GH millirobots in living mice: biochemistry analysis. The serum samples were collected for biochemistry analysis at 14th day, (n=4/group). Data are presented as the means ± s.e.m.

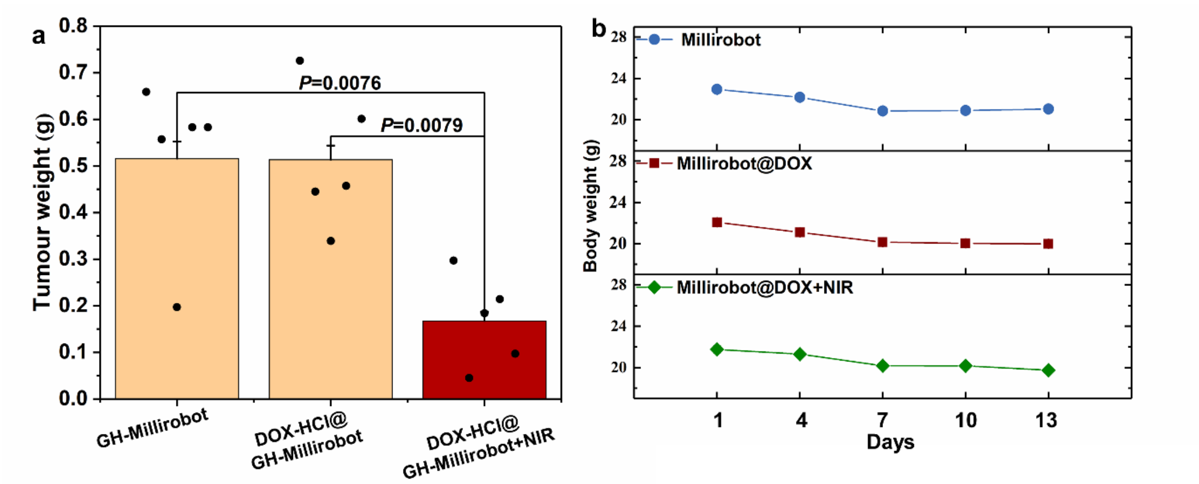

Fig. S16. Mice Tumor Therapy. a, Gastric tumor weight of mice in different groups, (n=5/group). Data are presented as the means ± s.e.m. **b,** Body weight of mice during the tumor treatment, (n=5/group).

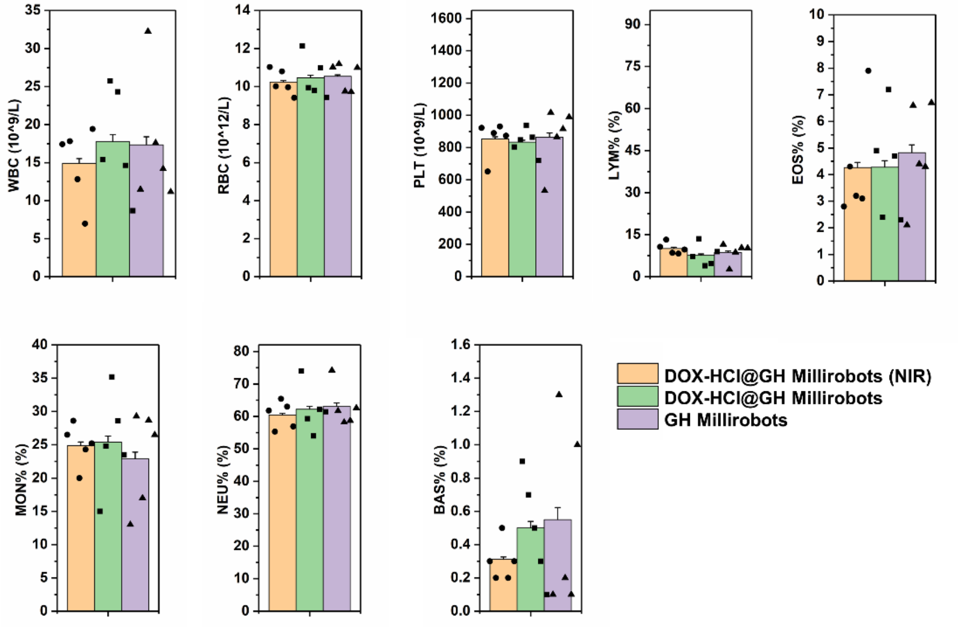

Fig. S17. Routine hematological analysis of mice tumor therapy. Whole blood was collected for routine hematological analysis at 14th day which is the end of treatment, (n=5/group). Data are presented as the means ± s.e.m.

Table S1. Comparison of swimming speeds and lateral drift rates of millirobots of conventional materials

Materials	Swimming velocity (body length/s)	Drift rate (%)	Ambient fluids	Magnetic field frequency (Hz)	Magnetic field strength (mT)	Ref.
Graphene/Ni	1.433	0.024	DI water	10	12	This work
Biohybrid/Fe_3O_4	0.556	0.441	DI water	10	7	[3]
Zirconium-silicon hybrid sol-gel material/Ni/Ti	0.960	0.240	DI water	10	1	[16]
Photoresist/Ni/Au	0.884	0.386	DI water	10	3	[28]
Hydrogel/Fe	1.433	0.54	DI water	10	25	[29]
SiO_2/Ni	0.712	0.140	DI water	10	8	[30]
Biohybrid/CuS/Fe_3O_4	1.318	0.515	DI water	10	10	[40]
Fe	0.442	0.202	DI water	10	10	[48]
Hydrogel/Fe_2O_3	0.550	1.000	DI water	10	10	[49]
C/Ni/Au	0.527	0.218	DI water	10	5	[50]
polymeriaing resist/Fe_2O_3/Au	0.316	0.26	DI water	10	20	[51]

Note that we used a higher magnetic field strength as compared to some other works because of the low loading of the magnetic metal particles in the GH millirobots (i.e., the magnetic metal Ni layer taking only 6% volume fraction). The low loading ensures the full suspension mode of the of millirobots and thus enables fast movement and precise trajectory control; however, due to the reduced magnetic content, the magnetic field strength has to be increased.
