# Supplementary material for: Lightweight and drift-free magnetically actuated millirobots via asymmetric laser-induced graphene
Source: Nat Commun. 2024 May 21;15:4334. doi: 10.1038/s41467-024-48751-x (PMC11109242; doi:10.1038/s41467-024-48751-x)
Supplement: Supplementary file 3 — Description of Additional Supplementary Files [file 41467_2024_48751_MOESM3_ESM.pdf]

Description of Additional Supplementary Files

File Name: Supplementary Video 1
Description: Fabrication process of porous helical LIG sheets

File Name: Supplementary Video 2
Description: Tracjectory of on-ground and suspending millirobot

File Name: Supplementary Video 3
Description: Actuation of GH millirobot with different chirality

File Name: Supplementary Video 4
Description: GH millirobot movement in the rat’ stomach

File Name: Supplementary Video 5
Description: Movement of the GH millirobot inside an isolated pig bladder
