# Supplementary material for: Lightweight and drift-free magnetically actuated millirobots via asymmetric laser-induced graphene
Source: Nat Commun. 2024 May 21;15:4334. doi: 10.1038/s41467-024-48751-x (PMC11109242; doi:10.1038/s41467-024-48751-x)
Supplement: Supplementary file 9 — Reporting Summary [file 41467_2024_48751_MOESM9_ESM.pdf]

nature portfolio

Corresponding author(s): Xin Chen, Yu-Jing Lu, Ni Zhao

Last updated by author(s): Apr 28, 2024

Reporting Summary

Nature Portfolio wishes to improve the reproducibility of the work that we publish. This form provides structure for consistency and transparency in reporting. For further information on Nature Portfolio policies, see our Editorial Policies and the Editorial Policy Checklist.

Statistics

For all statistical analyses, confirm that the following items are present in the figure legend, table legend, main text, or Methods section.

n/a	Confirmed	
☐	☒	The exact sample size (*n*) for each experimental group/condition, given as a discrete number and unit of measurement
☐	☒	A statement on whether measurements were taken from distinct samples or whether the same sample was measured repeatedly
☒	☐	The statistical test(s) used AND whether they are one- or two-sided<br>*Only common tests should be described solely by name; describe more complex techniques in the Methods section.*
☒	☐	A description of all covariates tested
☒	☐	A description of any assumptions or corrections, such as tests of normality and adjustment for multiple comparisons
☐	☒	A full description of the statistical parameters including central tendency (e.g. means) or other basic estimates (e.g. regression coefficient) AND variation (e.g. standard deviation) or associated estimates of uncertainty (e.g. confidence intervals)
☒	☐	For null hypothesis testing, the test statistic (e.g. *F*, *t*, *r*) with confidence intervals, effect sizes, degrees of freedom and *P* value noted<br>*Give P values as exact values whenever suitable.*
☒	☐	For Bayesian analysis, information on the choice of priors and Markov chain Monte Carlo settings
☒	☐	For hierarchical and complex designs, identification of the appropriate level for tests and full reporting of outcomes
☒	☐	Estimates of effect sizes (e.g. Cohen's *d*, Pearson's *r*), indicating how they were calculated

Our web collection on statistics for biologists contains articles on many of the points above.

Software and code

Policy information about availability of computer code

Data collection	Excel
Data analysis	MATLAB R2022a, Origin 2017 64Bit

For manuscripts utilizing custom algorithms or software that are central to the research but not yet described in published literature, software must be made available to editors and reviewers. We strongly encourage code deposition in a community repository (e.g. GitHub). See the Nature Portfolio guidelines for submitting code & software for further information.

Data

Policy information about availability of data

All manuscripts must include a data availability statement. This statement should provide the following information, where applicable:

- Accession codes, unique identifiers, or web links for publicly available datasets
- A description of any restrictions on data availability
- For clinical datasets or third party data, please ensure that the statement adheres to our policy

The data generated in this study are provided in the Supplementary Information/Source data file.   Source data are provided with this paper.

Research involving human participants, their data, or biological material

Policy information about studies with human participants or human data. See also policy information about sex, gender (identity/presentation), and sexual orientation and race, ethnicity and racism.

Reporting on sex and gender	N/A
Reporting on race, ethnicity, or other socially relevant groupings	N/A
Population characteristics	N/A
Recruitment	N/A
Ethics oversight	N/A

Note that full information on the approval of the study protocol must also be provided in the manuscript.

Field-specific reporting

Please select the one below that is the best fit for your research. If you are not sure, read the appropriate sections before making your selection.

☒ Life sciences ☐ Behavioural & social sciences ☐ Ecological, evolutionary & environmental sciences

For a reference copy of the document with all sections, see nature.com/documents/nr-reporting-summary-flat.pdf

Life sciences study design

All studies must disclose on these points even when the disclosure is negative.

Sample size	No statistical analysis was used to predetermine sample sizes. Sample sizes were decided based on the universal standards in the field. For in vitro analysis, at least 3 biological replicates were used for experiments. For immunofluorescence assay, the experiment was repeated three times independently with similar results. For animal experiments, 8 mice were included in each group.
Data exclusions	For in vitro tests, data that were drastic outliers due to technical variability were excluded. For animal samples, no data was excluded.
Replication	Experiments in this study were independently repeated at least three times, the numbers of replicates for each experiment were presented in the Figure legend. We confirm that all attempts at replication were successful.
Randomization	For animal samples, all samples were randomly numbered and deallocated into groups. For in vitro experiments, allocation of the different experimental treatments occurred randomly. In brief, cells were plated and plates were randomly assigned to experimental or control groups.
Blinding	For drug treatment experiments the investigators were not blinded as these experiments required the investigators to be aware of the grouping. However, the person who perform measurements was not informed about the goals of the study and the nature of the treatment groups.

Reporting for specific materials, systems and methods

We require information from authors about some types of materials, experimental systems and methods used in many studies. Here, indicate whether each material, system or method listed is relevant to your study. If you are not sure if a list item applies to your research, read the appropriate section before selecting a response.

Materials & experimental systems

n/a	Involved in the study	
☒	☐	Antibodies
☐	☒	Eukaryotic cell lines
☒	☐	Palaeontology and archaeology
☐	☒	Animals and other organisms
☒	☐	Clinical data
☒	☐	Dual use research of concern
☒	☐	Plants

Methods

n/a	Involved in the study	
☒	☐	ChIP-seq
☒	☐	Flow cytometry
☒	☐	MRI-based neuroimaging

Eukaryotic cell lines

Policy information about cell lines and Sex and Gender in Research

Cell line source(s)	SGC-7901 gastric cancer cells were obtained from iCell Bioscience Inc (Shanghai, China, Item No. icell-h277).
Authentication	DNA was extracted with Axygen's genome extraction kit, amplified by 21-STR amplification protocol, and the STR locus and sex gene Amelogenin were detected on ABI 3730XL genetic analyzer. A basically matched cell line was found in the cell line search. EXPASY database showed that the cell name was SGC-7901 and the cell number corresponded to CVCL_0236. Multiple alleles were found in this cell line.
Mycoplasma contamination	The cell line was tested negative for mycoplasma using the Sigma LookOut Mycoplasma PCR Detection Kit (#MP0035-1KT).
Commonly misidentified lines (See ICLAC register)	None

Animals and other research organisms

Policy information about studies involving animals; ARRIVE guidelines recommended for reporting animal research, and Sex and Gender in Research

Laboratory animals	8-week-old SPF SD rats and 5-week-old SPF female BALB/c nude mice were obtained from Charles River Laboratories (Beijing). The temperature and humidity of the feeding environment meet the standard of ordinary animal house, and keep the temperature and humidity at 20~26 °C, 40~70%. The alternating time of day and night 12h/12h.
Wild animals	This study did not involve wild animals.
Reporting on sex	This study did not involve reporting on sex.
Field-collected samples	This study did not involve field-collected samples.
Ethics oversight	This experiment was performed in accordance with the requirements of the international accreditation code of Association for Assessment and Accreditation of Laboratory Animal Care International (AAALAC) and the policies of the Institutional Animal Care and Use Committee (IACUC) (No.B202309-5).

Note that full information on the approval of the study protocol must also be provided in the manuscript.

Plants

Seed stocks	N/A
Novel plant genotypes	N/A
Authentication	N/A
